# Motivational factors in makerspaces: a mixed methods study of elementary school students’ situational interest, self-efficacy, and achievement emotions

**DOI:** 10.1186/s40594-018-0129-0

**Published:** 2018-11-30

**Authors:** Vanessa W. Vongkulluksn, Ananya M. Matewos, Gale M. Sinatra, Julie A. Marsh

**Affiliations:** 1 The Ohio State University, 29 West Woodruff Avenue, Columbus, OH 43210 USA; 2 Temple University, 1301 Cecil B. Moore Ave., Philadelphia, PA 19122 USA; 3 University of Southern California, 3470 Trousdale Pkwy, Los Angeles, CA 90089 USA

**Keywords:** STEM interest, Motivation, Self-efficacy, Achievement emotions, Design-based learning, Makerspaces

## Abstract

**Background:**

Design-based learning and makerspace programs have been shown to be effective in increasing student motivation for STEM learning. Since these programs have largely been implemented for middle school and older students, less is known about their motivational implications in elementary school contexts. The purpose of this study was to understand how elementary school students’ (grades 3–6) self-efficacy changed throughout the semester of a design-based makerspace course, and how these changes are associated with experiences of positive and negative achievement emotions. Additionally, this study investigated how self-efficacy and achievement emotions are related to students’ interest development in the makerspace course.

**Results:**

Results of hierarchical growth modeling showed that although students’ self-efficacy and situational interest remained moderately high during the course, both declined over the makerspace semester. Further, self-efficacy, as well as experiences of excitement and frustration with project tasks were found to be associated with students’ situational interest. Interpretive analysis of student think-aloud interviews and classroom observations supported these findings.

**Conclusions:**

Design-based makerspaces have the potential to trigger elementary school students’ interest in STEM activities. However, the iterative design process can lead to suboptimal outcomes on students’ self-efficacy and interest. Instructors should offer context-sensitive efficacy- and emotion-related scaffolds to foster positive makerspace experiences.

In recent years, the focus of the educational community has shifted towards helping students gain twenty-first century skills that lead to success in higher education and the workplace. One important focus of this movement has been on helping children develop complex problem-solving skills. These are precisely the skills students need to thrive in a world where knowledge of how to integrate cognitive, social, and communication skills are in demand (NRC 2010). Concurrently, education scholars and practitioners have sought avenues to increase students’ interest in STEM amidst concerns for the inadequacy of the STEM workforce to support growing US needs (NRC 2010; Sadler et al. 2012). Design-based learning (DBL) has emerged as a possible instructional strategy that could be the solution to both of these current educational issues, offering the potential to support the development of problem-solving skills and STEM interest.

Correspondingly, DBL programs have proliferated throughout the USA in the last few years, and research into these programs has pointed to their benefits. For example, DBL programs have been shown to be effective not only in increasing STEM content knowledge, but also competencies central to problem-solving such as critical thinking and self-directed learning (Choi et al. 2014; Doppelt et al. 2008; Loyens et al. 2008). However, empirical support for their additional motivational benefits are largely lacking. It remains unclear whether and how DBL programs might work to improve students’ interest. Also, much of the research in support of DBL has been conducted at the middle school, high school, and undergraduate levels, and few have examined DBL in the elementary school context.

The purpose of this study is to fulfill the aforementioned research gap by examining motivational variables related to students’ situational interest in a DBL course. Specifically, we sought to understand how students’ self-efficacy and situational interest changed throughout the semester during which a DBL course took place. The present study also investigated how students’ experiences of achievement emotions influence these motivational trends. We situate our mixed-methods research within a design-based makerspace course in an elementary school setting, with students grades 3 through 6.

## Design-based learning and makerspaces

Design-based instruction aims to increase students’ reasoning skill and transfer of content knowledge by engaging students in designing artifacts that solve real world problems (Doppelt et al. 2008). Although design-based instruction is known by various names, including design-based learning (DBL; Fortus et al. 2004; Gerber et al. 2012; Ke 2014; Mehalik et al. 2008; Doppelt et al. 2008), learning by design (LBD; Kolodner et al. 2003), problem-based learning (PBL; Blumenfeld et al. 1991; Dunlap 2005), and design-thinking instruction (Brown 2008), the core components remain relatively consistent. The design process within DBL and related programs generally consists of six stages: (1) learning about the design context, identifying needs, and defining the problem; (2) collecting information and gaining knowledge needed for design; (3) brainstorming and creating multiple alternative solutions; (4) choosing the optimal solution based on needs and contextual limitations; (5) constructing the prototype of design ideas; and (6) sharing prototypes with others (including intended users) for evaluation and modifying the design based on feedback (Brown 2008, Doppelt and Schunn 2008; Mehalik et al. 2008.)

DBL is a unique instructional method because, unlike most academic tasks, it requires students to solve ill-structured problems. Ill-structured problems are context-specific problems in which the problem situation is not well-defined, the problem description not clearly laid out, and the information necessary to solve the problem is not given in the problem statement (Jonassen 1997). At the beginning of their design projects, students need to conduct research to define their problem situation and find the information needed to lay out their design plan. Jonassen (1997, 2000) referred to this phase as “problem representation” and “searching for solutions.” Students need to define the “problem space” by mapping the problem onto prior knowledge, decomposing the problem into stages or component parts, and generating possible ways to solve each component part to reach the solution state. As such, DBL promotes important processes of learning, such as linking newly learned content to prior knowledge and transferring what is learned to solve new problems. These learning activities also promote critical thinking and creativity, as students are asked to come up with their own, novel solutions to a real-world problem.

Makerspaces, an instructional method related to DBL, have also grown in popularity in recent years, particularly in informal learning sites outside the classroom. Compared to DBL, makerspaces as a relatively new instructional medium have a less consistent definition. Sheridan et al. (2014) defined makerspaces as “informal sites for creative production in art, science, and engineering where people of all ages blend digital and physical technologies to explore ideas, learn technical skills, and create new products” (p. 505). With this definition, makerspaces are broadly defined to include such making spaces in museums and libraries, which typically lack clear academic goals. We propose the use of the term “design-based makerspaces” to refer to makerspaces structured around DBL components mentioned above. This definition combines the clear design goals associated with DBL and the STEM-focused creative production emphasized in informal makerspaces. This new term is also useful as makerspaces are increasingly considered in K-12 settings, prompting the need for a more well-defined making and designing process compared to informal makerspaces.

## DBL and learning outcomes

Makerspaces have not been the focus of education research despite its rising popularity. Because our particular research context focused on a design-based makerspace program, with design stages similar to other DBL programs, we examined the DBL literature base to discern the learning and motivational processes that may have been present in our context.

The design process involves learning content knowledge needed for design, and adapting such knowledge to fit real-world design needs throughout the iterative evaluation and modification processes. As such, studies have shown that DBL programs are successful for delivering STEM concepts. At the high school and undergraduate levels, students in DBL programs were successful in transferring content knowledge from fields of engineering (Gerber et al. 2012), science (Kolodner et al. 2003; Doppelt 2009), and graphic design (Fontaine 2014). At the middle school level, students also demonstrated the use of science (Doppelt et al. 2008; Mehalik et al. 2008) and math (Ke 2014) concepts in designing their artifacts. Students in these programs showed gains in content knowledge according to analyses of their pre- and post-program scores in knowledge domains related to their design projects. In addition, these gains in content knowledge via DBL instruction were greater when compared to control students in the scripted inquiry condition (Mehalik et al. 2008). Education scholars have contended that gains in content knowledge from DBL experiences are more meaningful compared to traditional STEM instruction. Students are asked to transfer content to solve open-ended, authentic problems rather than closed-ended, simulated problems as is the case in traditional instruction (Doppelt 2009).

Another positive facet of DBL is its capability to motivate students towards STEM learning. One important component of DBL is the provision of student autonomy in terms of deciding which design project to take on, determining the content knowledge needed to complete design tasks, and establishing a work plan that feasibly allows for on-time completion (Doppelt 2009; Doppelt et al. 2008). DBL programs are postulated to increase student motivation for STEM learning because of the explicit application of content knowledge to real-world situations and the sense of personal ownership students feel throughout the design process. Relatedly, design-based instruction has been shown to be effective in increasing students’ interest and engagement in science and math classes (Doppelt and Schunn 2008; Doppelt et al. 2008; Gerber et al. 2012; Ke 2014).

## DBL, self-efficacy, and achievement emotions: a social cognitive theory perspective

In addition to supporting STEM learning and offering a motivating learning context, another key incentive for DBL’s adoption is its potential for increasing students’ active choice to participate in STEM-related tasks. Our study used social cognitive theory in framing how motivational variables interact to influence students’ academic choices (Bandura 2001; Bandura and Schunk 1981). Social cognitive theory posited a framework of triadic reciprocality. Triadic reciprocality is the concept that environmental variables, personal factors such as cognition and motivation, and behaviors are related in a pattern of reciprocal interactions. Individuals’ cognitive and motivational processes contribute to their decisions to participate in specific tasks, which in turn influence their environments and behaviors. After participation in academic activities, students self-reflect and process new information about consequences of actions, perceived capabilities, and outcome expectations (Bandura 2001). Therefore, engaging in STEM tasks such as design-based makerspaces contribute to students’ sense of proficiency with STEM activities and decision to participate in similar tasks in the future.

The process of self-reflection is intertwined with students’ perception of self-efficacy, a key motivation variable (Bandura 2001). Self-efficacy is defined as “beliefs in one’s capabilities to organize and execute the courses of action required to produce given attainments” (Bandura 1997, p. 3). As a motivation variable, self-efficacy represents how individuals evaluate their ability (via self-reflection) and decide whether or not to participate in a specific action. In the context of design-based makerspaces, self-efficacy refers to students’ belief about their ability to successfully complete a design project that is effective, at least conceptually, in solving the identified problem.

Self-efficacy is informed by four sources of information: mastery experience, vicarious experience, verbal persuasion, and physiological or emotional reaction (Bandura 2001). The source of self-efficacy our study focused on is the emotional reaction towards a task, (i.e., the makerspace project), which in a learning context is closely related to achievement emotions (Pekrun 2006).

Achievement emotions can be categorized as either related to the activity itself or to task outcomes (Pekrun 2006). Activity-related emotions, such as excitement or boredom, are those that are experienced during task engagement. Outcome-related emotions, such as hope or anxiety, depend on the nature of task outcomes as perceived by students. Our study focused on activity-related emotions because we are interested in examining achievement emotions students experienced during the makerspace course. Valence and activation are two additional dimensions of classification (Linnenbrink-Garcia and Pekrun 2011). Valence ranges along an axis of positive to negative emotional states. Emotions such as excitement are positive emotions, whereas those such as frustration are negative emotions. States of activation refer to whether the emotion tended to spur or suppress cognitive activity. For example, activation states for positive emotions range from excitement as an activating emotion to relaxation as a deactivating emotion.

When students participate in an academic task, they appraise emotional reactions as part of task outcomes. Thus, emotional reactions inform efficacy beliefs. Scherer (Scherer et al. 2001), Pekrun (2006), Goetz (Goetz et al. 2010) and their associates further described that the relationship between achievement emotion and self-efficacy is related to students’ perception of academic control. Achievement emotions are triggered when students varyingly appraise how much control they have as well as the importance of the task they have to perform (Pekrun 2006). When participating in a task for which they perceive high levels of control and competence, students expect more positive outcomes, resulting in positive emotions such as excitement and pride. In contrast, students who participate in a task for which they perceive low levels of control have negative outcome expectations, leading to negative emotions such as frustration and anger. These emotions, in turn, become part of students’ self-reflection process. They inform ability beliefs and influence future actions as students endeavor to avoid negative performances and emotional outcomes in the future.

The theoretical relationship between achievement emotions and self-efficacy have found numerous empirical support, with positive emotions such as excitement and pride found to be positively related and negative emotions such as frustration and anger negatively related to ability beliefs (Goetz et al. 2008; Goetz et al. 2006; Pekrun et al. 2006; Gumora and Arsenio 2002). In addition to these directional relationships, Goetz and associates (2010) posited that the “strength of relation” should also be considered when examining associations between ability beliefs and emotions (p. 46). Domain general ability perceptions such as academic self-concept is mainly focused on outcomes (i.e., students who assess that they perform well academically would have high academic self-concept). Therefore, academic self-concept has stronger relations with outcome-related emotions (Goetz et al. 2010; Pekrun et al. 2002). In contrast, self-efficacy is more context- and domain- specific, and therefore has stronger relations with activity-related emotions. Applied to the context of our study, we could expect that students’ self-efficacy as appraised within a context-specific makerspace setting would be more strongly associated with activity-related achievement emotions such as excitement and frustration, rather than to outcome-related emotions such as pride or disappointment.

## DBL and interest development

Following social cognitive theory, students’s self-efficacy is posited to be closely associated with their interest for specific academic activities. According to Bandura and Schunk (1981), a requisite level of self-efficacy is needed for students to develop intrinsic interest in an academic task or task domain. They also suggested that students need to build up a series of mastery experiences that increase their self-efficacy before interest towards an activity can be developed. Therefore, there may be a “temporal lag” between demonstrable increases in self-efficacy and increases in intrinsic interest (p. 597). Numerous studies have contributed empirical support for the close, contemporaneous association between self-efficacy and interest (Cordova et al. 2014; Wigfield et al. 1997; Lee et al. 2014). However, others have found null results between self-efficacy increase and interest change, particularly in intervention settings where self-efficacy increase was induced through a systematic intervention plan (Bailey et al. 2017; Bandura and Schunk 1981). These mixed results suggest that the “temporal lag” between self-efficacy increase and interest change posited by Bandura and Schunk (1981) may not occur consistently across contexts and require additional investigation.

In addition to social cognitive theory, we also drew upon a prominent theory of interest development by Hidi and Renninger (2006). They proposed that interest develops through four phases including: (1) triggered situational, (2) maintained situational, (3) emerging individual, and (4) well-developed individual interest. We focused on how students develop triggered and maintained situational interest based on experiences with their design projects, as well as how achievement emotions were related to their developing interest.

### Triggered situational interest

In the first phase, interest is “triggered” by specific characteristics of stimuli such as personal relevance, humor, and novelty (Sadoski 2001; Renninger and Hidi 2002). Triggered interest may also occur when learners encounter instructional activities that provide developmentally appropriate challenge and intensity. In this first phase, learners often demonstrate fleeting attention to content and higher levels of affect (both positive and negative emotions). For example, an activity that makes students either excited or frustrated may trigger their interest. This first phase of interest development is necessary to allow for more lasting individualized interest development (Renninger et al. 2008). However, although students may have their interest initially hooked, this interest may be temporary without further autonomy support, guidance, and opportunity for follow up exploration (Renninger and Su 2012).

### Maintained situational interest

Maintained situational interest involves focus, attention, and persistence, which may reoccur over an extended period of time (Renninger and Su 2012). The more personally relevant and meaningful tasks are, the more likely it is for interest to be maintained (Harackiewicz et al. 2000). Connecting instructional materials to students’ prior knowledge and experiences is essential to fostering personal relevance and meaning. Personal relevance for academic tasks is also linked to high task value, both of which are critical to replacing initial negative reactions that may have been triggered with positive emotions. Fostering positive emotions is crucial for triggered situational interest to turn into maintained interest. Since design-based makerspaces aim to increase personal relevance and task value by asking students to apply STEM concepts to solve self-identified problems, we expect that students will develop both triggered and maintained situational interest in STEM content related to their design projects.

## The present study

Although DBL programs and makerspaces are touted for their motivational benefits, few studies have directly examined how DBL are related to motivational outcomes. Our objective was to gain insights into how participation in a design-based makerspace course influenced students’ situational interest, self-efficacy, and achievement emotions. Further, questions remain about how best to tailor DBL programs for different student populations to support positive self-efficacy beliefs and interest. In particular, educational scholars have voiced concerns about design-based instruction and its suitability for students who have lower levels of scientific knowledge related to the design task. For example, the open-ended nature of the design process may be too difficult for low-performing or younger students who are still grappling with understanding basic STEM concepts (Doppelt et al. 2008). Students who lack a strong background knowledge in STEM are more likely to have low self-efficacy in learning and using STEM concepts, and may have less confidence in executing design tasks. This lack of confidence is especially detrimental when the design process calls for a high degree of self-initiation and self-regulation. Students’ lack of knowledge could also lead to feelings of frustration, which further undermine self-efficacy and interest development. Despite these context-specific concerns, few existing studies have examined the motivational processes occurring in DBL programs for young students in an elementary school context. Relatedly, the role of important factors such as self-efficacy and achievement emotions in interest development within this setting remains unclear.

The purpose of the present study was to explore the motivational processes that occur during a design-based makerspace program, particularly one in an elementary school context. Specifically, we sought to understand how students’ self-efficacy changes throughout the semester of the design-based makerspace course, and how these changes are associated with experiences of positive and negative achievement emotions. Additionally, we aimed to investigate how situational interest changed throughout the makerspace course, and how these changes are associated with self-efficacy and achievement emotions. The research questions guiding the study are the following:

1. Does students’ self-efficacy change throughout a design-based makerspace course situated in an elementary school context?
  - Is students’ self-efficacy associated with achievement emotions experienced during the design process?
  - Is students’ self-efficacy trajectory associated with achievement emotions?
2. Does students’ situational interest in the makerspace course change throughout course?
  - Is students’ situational interest associated with their self-efficacy and achievement emotions?
  - Is students’ situational interest trajectory associated with their self-efficacy and achievement emotions?

## Methods

### Participants and context

Participants were students (3rd–6th grade; *N* = 100) who attended a design-based makerspace course as part of their regular school experience in Spring 2016. The course was offered at a private elementary school in Southern California to all students grades 3–6. Unfortunately, students’ ethnic information was not collected as part of the study. However, we expected that participants’ ethnic makeup reflects the demographics of the school’s population, given grade-wide participation in our study. Specifically, the school’s population consisted of 69% White-Caucasian students, 10% African American, 6% Asian or Pacific Islander, 5% Hispanic, 10% two or more ethnicities, and less than 1% other ethnicity.

The private school in which the research took place housed students who came from primarily affluent families. Although scholarships were available, most families paid a tuition of more than $20,000 per year for their child to attend this school. The school is nationally recognized for the strong performance of their students and innovative usage of technology. Students are exposed to digital technology from pre-kindergarten, and graduate to 1:1 access to laptops beginning from third to sixth grade. At the time of the research, administrators introduced the makerspace program as a way to combine the school’s focus on student-led inquiry with technology-rich instruction. Students benefit from a wealth of digital and traditional resources for their makerspace projects, including a computer lab, 3D printer, green screen audio-visual room, and dedicated makerspace area complete with hand tools.

This semester-long course was structured based on design-based learning components, requiring students to identify a real-world problem and design a product or prototype that addresses it using STEM concepts. Students were given quite a bit of freedom in selecting their projects. During the fall semester prior to the beginning of the research study, students attended pre-makerspace sessions to identify projects they would like to work on in the spring semester. Teachers asked students to identify an inquiry question shaped by a real-world need. An example inquiry question for a 4th grade boy was, “How might I learn to make a movie to help people who are hospitalized stay positive and lift their spirits?” Another boy from sixth grade asked, “How might I learn to 3D model/print to help children without limbs do everyday activities without having to replace their prosthetics?” Teachers clearly scaffolded this process, and all students came up with an inquiry question using the same structure. Importantly, when deciding on feasible projects, students were not limited by the tools already present at school. They could request for additional purchases (within reason) of other materials that their project required. For example, a number of students ordered electronic circuit kits such as Arduino and LittleBits for use on their projects. The freedom students had in choosing projects was taken a step further with the relatively unrestrained access to resources.

During the makerspace semester (Spring 2016), students had the option to work in teams of two, or by themselves on their design projects. Teachers and mentors guided them through the iterative process of creating and evaluating their design products. There were two makerspace classes organized according to grade level. The lower-level class consisted of 3rd and 4th grade students, and the higher-level class consisted of fifth and sixth grade students. Students in each makerspace class shared work areas, resources, and mentors according to their project type. For example, students in the lower-level class who worked on computer-related projects spent their makerspace hour with each other and their project mentors in the computer lab. The time and space logistics were similar for fifth and sixth grade students in the higher-level class.

Students came up with a range of design products. The student mentioned above who wanted to make a movie to help hospitalized patients ended up writing a script for a funny short movie, recruiting other students to act out scenes in front of a green screen, and using a computer software to add animation effects and edit the movie. The other sixth grade boy who wanted to design an adjustable prosthetic arm sketched out his prosthetic design, deconstructed this sketch into several parts that he can print using a 3D printer, transferred his drawings into a 3D printing computer program, and assembled a model of a prosthetic arm that could be adjusted around the elbow to automatically resize as the user grows older.

### Measures

#### Self-efficacy

Five items measuring self-efficacy were adapted from guidelines specified by Bandura 2006; *α* = 0.73–0.82 across time points). All items for this study were measured on a 5-point Likert scale, using emoticons to support students’ understanding of the survey (Danielson et al. 2015). Self-efficacy items were measured using a scale with 1 = No to 5 = Yes. A sample item is, “I expect to do very well on my makerspace project.”

#### Situational interest

Seven interest items were adapted from Danielson et al. (2015; *α* = 0.86–0.91 across time points). Items were measured on a 5-point Likert scale (1 = No to 5 = Yes), measuring the extent to which students enjoyed makerspace activities as well as whether they would choose to do makerspace activities in their spare time. A sample item is, “If you have free time outside of school, would you do the type of activities you do during makerspace time?”

#### Achievement emotions

Four activity-related achievement emotion items were adapted from Pekrun et al. (2011), measuring the extent to which students experienced frustration, confusion, excitement, and curiosity while conducting tasks in the makerspace course. Items were measured on a Likert scale (e.g., 1 = not frustrated to 5 = very frustrated). A sample item is, “How frustrated do makerspace activities make you?”

#### Demographics

Students’ makerspace class level (0 = lower-level class, 1 = higher-level class) and gender (0 = male, 1 = female) were also collected as part of the survey.

### Data collection

Students were given surveys during their makerspace class at three time points in the semester. At the beginning of the semester, students took the self-efficacy and demographic survey before beginning their makerspace project. The mid-semester and end-of-semester surveys included items for self-efficacy and situational interest. The mid-semester survey also included items that tapped students’ experiences of achievement emotions. Researchers were present during all survey administrations to answer any questions. Students took about 15 min to complete the survey, on average.

We also collected qualitative data to provide further evidence, depth, and complexity of student experiences in order to triangulate our quantitative findings. We conducted three classroom observations for each class level and took semi-structured field notes. Within our observation field notes, we focused on students’ interactions with their teammates if they were working in pairs, their interactions with mentors, the tools they were using, and the state of their design products. Additionally, we conducted three interviews (i.e., think-alouds) with each student or student team over the course of the semester (conditional on parental consent, *n* = 70). Think-alouds were scheduled at the beginning, middle, and end of the semester. Each think-aloud interview lasted 5–10 min and were conducted as students were engaging in the design and construction processes. These think-alouds allowed us to gather real-time information on student’s emotions, interest, and efficacy. Although these interviews were largely unstructured conversations, we did have prompts such such as, “How do you feel about your project today?” or “Tell me about your progress on this project.” Interviews were audio-recorded and transcribed by the researchers.

### Data analysis

#### Quantitative analysis

To answer the first research question regarding self-efficacy change, piecewise, two-level growth models of self-efficacy were fitted within the hierarchical linear modeling (HLM) framework, with time specified at level 1 and student at level 2. This specification accounted for residual correlations and heteroskedasticity resulting from intra-individual nesting effects (Raudenbush and Bryk 2002). It addressed the expectation that motivation variables measured from the same student over time will be more similar to one another compared to those of other students. In addition, due to the nature of this specific makerspace context, heteroskedasticity may also be present for students in the same makerspace class because students in each class shared resources and attended makerspace class during the same time of the day. These similarities are expected to translate to more similar trends in the motivational variables measured. To account for this pattern, the specified models allowed residual variance to differ between classes (Rabe-Hesketh and Skrondal 2012). Specifically, an independent correlation structure was specified, resulting in the estimation of separate variance parameters for each makerspace class.

The piecewise growth models included two slopes, one representing self-efficacy growth from the beginning of the semester to the middle of the semester, and another representing growth from the middle of the semester to the end of the semester. A total of four models were specified. The first was an unconditional model at level 2 (student level), which solely examined self-efficacy trends over time. The second model was a control variables only model, which examined the influence of makerspace class level and gender on self-efficacy trends. The third, full model added achievement emotion variables to examine how experiences of positive (excitement, curiosity) and negative (frustration, confusion) emotions during the makerspace program are associated with levels of self-efficacy at each time point (Question 1a). Finally, the interaction model added moderation effects between achievement emotion variables and the slope parameters to discern whether experiencing these emotions are associated with how self-efficacy changes over time (i.e., whether the time slope differed according to levels of achievement emotions; Question 1b).

To answer the second research question regarding the associations of self-efficacy and achievement emotions with situational interest, four hierarchical linear growth models were specified to represent changes in situational interest from the middle to the end of the makerspace semester. The first model was specified to be unconditional at level 2, and the second model included only demographic variables as covariates. The third, full model added a time-varying self-efficacy variable as a predictor, testing how contemporaneous self-efficacy perceptions are associated with corresponding changes in situational interest. Other predictors specified in the model included self-efficacy at the beginning of the semester (self-efficacy_0_), two negative achievement emotions, and two positive achievement emotions. Finally, the last model added interactions of achievement emotions and self-efficacy with the slope parameter to test whether these predictors are associated with how situational interest develop through the makerspace semester.

#### Qualitative analysis

The qualitative data analysis was a two-step process. First, we used descriptive coding (Saldaña 2013, p. 87) to categorize data by basic theoretical ideas that were relevant from our literature review. Our coding tree started with “self-efficacy,” “achievement emotions,” and “situational interest” as the major coding categories under which other codes emerged both from theory and the data itself. Under the “self-efficacy” codes, statements related to beliefs about progress and ability to complete tasks were all coded. We also coded statements related to effort as a sub-code under self-efficacy, because students’ self-reflections regarding their own effort were often tied to their sense of efficacy overall. Under the “achievement emotions” codes, we again sorted stated emotions as either positive or negative in valence, high or low in activation, and activity or outcome related. Finally, under the “situational interest” codes, we coded any statements related to “hooks” or triggers that described students’ initial topical attention, as well as statements regarding enjoyment, persistence, continuity, and overall engagement.

Second, in order to directly address the research questions, we used matrices (Miles et al. 2013) to sort the coded data by participants’ makerspace class level (low vs. high), and interview time point (early, middle, or end) to both account for differences in self-efficacy, emotions, and interest across the two groups, as well as to better see how the participant experiences shifted over the course of the semester. We used the matrices to identify potential patterns.

## Results

Table 1 presents scores for self-efficacy and situational interest across time points and grade levels. Both self-efficacy and interest levels were high in general across time points, remaining higher than the median score (i.e., three out of the possible five) for most students. Closer examination of these scores also suggest that students in the lower-level makerspace class tended to have higher self-efficacy and interest compared to students in the higher-level class. Descriptive results showed that self-efficacy and situational interest appeared to decrease as the semester progressed, and this decline is especially apparent for higher-level class students. Table 2 presents scores for four achievement emotions measured during the mid-semester data collection. Most students appeared to have more positive emotions than negative emotions as they participate in makerspace activities. Results also showed that students in the lower-level class experienced higher levels of positive emotions (i.e., excitement and curiosity) compared to older students. Younger students also reported lower levels of frustration compared to older students.

**Table 1:** Self-efficacy and interest scores across time points and grade levels

	Self-efficacy				Interest		
	Time_0_	Time_1_	Time_2_	Mean_0–2_	Time_1_	Time_2_	Mean_1–2_
Lower-level class (*n* = 47)	4.31 (0.64)	4.14 (0.71)	4.30 (0.79)	4.25	3.83 (0.75)	3.70 (1.08)	3.76
Higher-level class (*n* = 53)	4.15 (0.68)	3.94 (0.69)	3.66 (0.83)	3.92	2.99 (1.10)	2.32 (1.10)	2.65
Mean (across class level)	4.23	4.06	3.99		3.42	2.99	

Notes: raw mean score (standard deviation). Subscripts refers to data collection rounds; 0 = beginning of semester, 1 = mid-semester, 2 = end of semester

**Table 2:** Achievement emotion scores across time points and grade levels

	Positive emotions		Negative emotions	
	Excited	Curious	Frustrated	Confused
Lower-level class (*n* = 47)	3.75 (1.31)	3.23 (1.34)	1.82 (0.95)	1.80 (0.76)
Higher-level class (*n* = 53)	2.72(1.22)	2.74 (1.29)	2.46 (1.38)	1.98 (1.26)
Mean (across class level)	3.21	2.99	2.15	1.87

Notes: raw mean score (standard deviation). Achievement emotion scores are collected at the mid-semester data collection

Prior to the main analysis, Pearson’s correlations were calculated between the variables of interest, including self-efficacy, situational interest, and emotions (Table 3). Self-efficacy and situational interest at all time points were all mutually significantly correlated with one another (*p* < 0.05). Self-efficacy scores across time points were also significantly correlated with students’ experience of excitement, curiosity, frustration (except with SE_0_), and confusion. As expected, self-efficacy scores were positively correlated with positive emotion scores and negatively correlated with negative emotion scores. Similarly, situation interest across time points were also significantly correlated with excitement, curiosity, and frustration. Students’ experience of confusion was negatively correlated to interest at the end of the semester.

**Table 3:** Pearson’s correlations

	1.	2.	3.	4.	5.	6.	7.	8.	9.
1. Self-efficacy_0_	1								
2. Self-efficacy_1_	0.52***	1							
3. Self-efficacy_2_	0.35***	0.58***	1						
4. Interest_1_	0.39***	0.57***	0.55***	1					
5. Interest_2_	0.23*	0.35***	0.64***	0.71***	1				
6. Excited	0.40***	0.53***	0.54***	0.78***	0.71***	1			
7. Curious	0.22*	0.27**	0.36***	0.49***	0.40***	0.48***	1		
8. Frustrated	− 0.20	− 0.24*	− 0.29**	− 0.43***	− 0.44***	− 0.32**	− 0.13	1	
9. Confused	− 0.24*	− 0.35***	− 0.32**	− 0.18	− 0.23*	− 0.18	0.05	0.44***	1

Notes: subscripts refers to data collection rounds; 0 = beginning of semester, 1 = mid-semester, 2 = end of semester. Achievement emotion scores are from the mid-semester data collection

* *p* < 0.05; ** *p* < 0.01; ***p < 0.001

### Self-efficacy trajectory

#### Self-efficacy declined throughout the semester

**Fig. 1:**
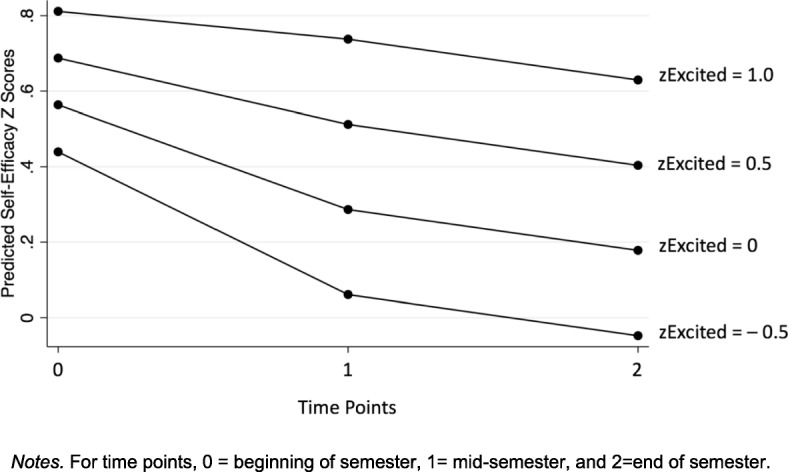
Moderation effect of excitement on self-efficacy trajectories

To answer the first research question on whether self-efficacy changed throughout the course, piecewise growth models of self-efficacy were fitted to examine students’ self-efficacy development across time. Standardized scores were used for self-efficacy and emotions, and as such emotion regression parameters could be interpreted as beta weights. Results from the unconditional model without level-2 covariates showed that students’ self-efficacy significantly decreased between the beginning and the mid-point of the semester (Table 4), but did not change significantly between the mid-point and the end of the semester. The control variables-only model indicated that self-efficacy at each time point was significantly associated with makerspace class level. Echoing descriptive trends, students in the higher-level class had significantly lower self-efficacy scores at each time point. The full model added emotions to the growth model and results showed that students’ experience of excitement was a positive predictor of self-efficacy, whereas confusion was a negative predictor. Finally, interaction effects between the four emotions and the slope parameters (Time_0–1_ and Time _1–2_) were fitted (not all shown in table). Only the interaction between excitement and the first slope parameter was statistically significant and was retained in the final interaction model. This result showed that students who felt more excited not only had higher self-efficacy scores at each time point, but also had a slower decline in self-efficacy between the beginning to the mid-point of the semester. For example, a student who has a standardized excitement score at the mean (zExcited = 0) is predicted to have a 0.28 standard deviation decrease in self-efficacy between the beginning and the middle of the semester. In contrast, a student who has a standardized excitement score one standard deviation above the mean (zExcited = 1) is predicted to only have a 0.08 standard deviation decrease in self-efficacy. This pattern is represented in Fig. 1, illustrating predicted self-efficacy scores across time points for students with differing levels of excitement, holding other emotion scores at the mean and demographic variables at zero (i.e., for male students in the lower-level class).

**Table 4:** Piecewise growth model of makerspace self-efficacy

	Unconditional model	Controls-only model	Full model	Interaction model
Time_0–1_	− 0.27* (0.11)	− 0.27* (0.10)	− 0.27* (0.11)	− 0.27* (0.10)
Time_1–2_	− 0.12 (0.11)	− 0.12 (0.11)	− 0.11 (0.11)	− 0.10 (0.11)
Excited	–	–	0.38*** (0.07)	0.25** (0.09)
Curious	–	–	0.09 (0.07)	0.09 (0.07)
Frustrated	–	–	− 0.01 (0.07)	0.01 (0.07)
Confused	–	–	− 0.23*** (0.07)	− 0.23** (0.07)
Excited * time_0–1_	–	–	–	0.20* (0.09)
Class level(Lower-level class = 0)	–	− 0.43** (0.15)	− 0.03 (0.05)	− 0.03 (0.14)
Gender (male = 0)	–	− 0.10 (0.16)	− 0.20 (0.13)	− 0.20 (0.16)
Intercept	0.48** (0.18)	0.83** (0.29)	0.77** (0.25)	0.56** (0.20)

Unstandardized regression coefficient (standard error)

Notes: subscripts refers to data collection rounds; 0 = beginning of semester, 1 = mid-semester, 2 = end of semester. Standardized scores were used for self-efficacy scores and achievement emotion scores

* *p* < 0.05; ** *p* < 0.01; ***p < 0.001

**Fig. 2:**
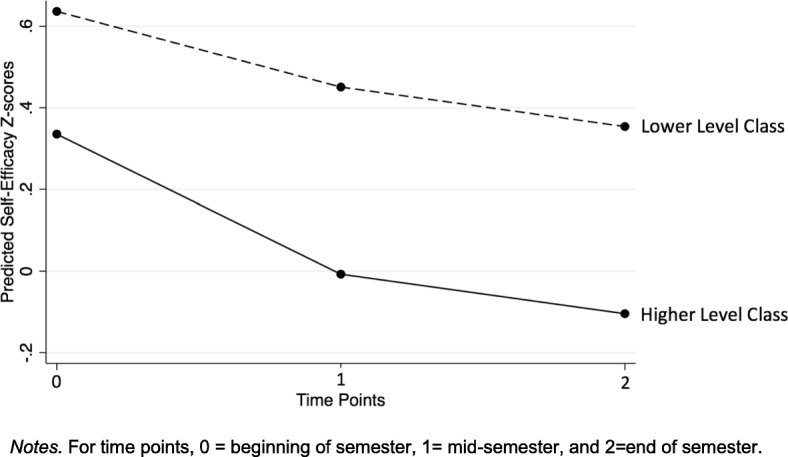
Predicted self-efficacy trajectories, by class level

To better understand the trajectory of self-efficacy within the specific makerspace context, we modeled changes in self-efficacy for an average student in each makerspace class level and graphed these predicted trajectories (i.e., holding other variables at grade-level means and specified male as the gender) (Fig. 2). The graph illustrates the general piecewise growth model result that there was a significant decrease in self-efficacy between the beginning to the middle of the semester, but relatively little change in self-efficacy between the middle and the end of the semester. However, the graph also shows that older students experienced a steeper decline in self-efficacy between the beginning and middle of the semester, which is related to these students experiencing less excitement on average compared to students in the lower-level class. Relatedly, similar to descriptive results, students in the lower-level class are shown to have higher self-efficacy overall compared to older students.

#### Students reappraised self-efficacy by mid-semester

In our qualitative data, we saw multiple student accounts of their self-efficacy illustrating a similar narrative to quantitative trends. In the early part of the semester, students in both class levels typically expressed high self-efficacy for successfully completing their design projects. As revealed in quotes from two typical students’ think-aloud responses (Table 5), students usually discussed plans for their project at the start of the semester. For example, a girl in the higher-level class who was making a virtual reality headset for hospitalized children expressed, “We’re just making it out of cardboard because I don’t think there is a kit. So, we will make it just for them” (student B, beginning of semester). Students are also typically focused on their assets at the beginning of the semester. For instance, another girl in the lower-level class expressed, “Me and my partner are both good at problem solving, and whenever one of us gets stuck, we help each other out” (student A, beginning of semester). The progress-focused thinking and positive evaluations of their ability suggest that that students tended to begin with high perceived self-efficacy to successfully complete their design projects.

**Table 5:** Exemplary student quotes demonstrating self-efficacy trends

Group level	Beginning of semester	Mid-semester
Student A (female/lower-level class)	This is a scale-down model - out of cardboard - of the final presentation hopefully. I sound crazy - but I just have to tape this piece here and it will work. I feel like coming up with the idea wasn't hard but we are the maker thinker mindset. Making stuff is not hard, our way of doing things isn't making a thousand prototypes, but to focus on one thing and make it really good. Me and my partner are both good at problem solving, and whenever one of us gets stuck, we help each other out.	This is our final stage - we have to assemble the wood and set up the electronics. The wood was cut to exactly what we needed, and hopefully the assembly will go well. When we problem solve we play around and talk amongst ourselves. Even though we do most of the work with our partners, I feel pretty confident working by myself.
Student B (female/higher-level class)	It took us like 3 weeks to make a model. With all the measurements. We're just making it out of cardboard because I don't think there is a kit…So we will make it just for them, instead of just making it plain.	This is a trial, kind of. We were planning to make it, but we didn't have enough time. We kind of want to build it, but our teachers said we couldn't make it in time. This (the first prototype) took us, like five weeks. [If we had more time we would] make the real model. [Our project] turned out different. When we first started, I thought we would make the real thing at the end, but in the end, we [will just] make the model. I thought you didn't have to do this much research. I thought we would work on this project for two or three weeks then move on to a different project. But, we have to research on this one thing and it took us a really long time.

At the mid-point of the semester, many students reevaluated what they could feasibly do for their projects given time and ability constraints, and generally showed a decreased sense of self-efficacy compared to the beginning of the semester. For example, a higher-level class boy who was designing a productivity app discussed picking a coding platform, “This is my interface. It was really hard…Coding does not come easy. My mentor suggested another platform called app inventor, which is basically like a strip down version for coding for android. And I was like, no. I can't do this. Xcode is definitely more for the specialist” (quote not shown in table). Like this student, many students admitted that working on their project was more difficult than they thought it would be. Part of the common difficulty was having to try out different ways to complete their design and changing course when something did not work. The student quoted above decided to use a more difficult coding platform because it allowed more flexibility in how he could design his app. In the course of weighing different options, students likely readjusted their self-efficacy and often had lowered ability perceptions as they encountered the difficult realities of their design projects.

Furthermore, students often expressed their frustration with the limited time they had to complete their projects at the mid-semester interviews. Student B, a girl in the higher-level class, told us, “This is a trial, kind of. We were planning to make it, but we don’t have enough time. We kind of want to build it, but our teachers said we couldn't make it in time. This (the first prototype) took us, like five weeks” (Table 5; student B, mid-semester). Another boy in the lower-level class who was making an Egyptian-themed game expressed, “I decided that I couldn't make the entire game. So, I’m just making one level” (quote not shown in table). These students were shown to be grappling with what they can realistically get done by the end of the semester, and were revising their design plans accordingly. By mid-semester, students’ emerging realization of the time constraint likely negatively influenced their self-efficacy to successfully complete their design projects.

Think-aloud interview data also showed differences in how students described their project’s progress by mid-semester. Echoing quantitative results, younger students typically have more positive evaluations of their progress and focused more on what still needed to be done. For example, Table 5 displayed example quotes from lower- and higher-level class students demonstrating this trend. The younger student discussed the remaining steps that need to be taken (“we have to assemble the wood and set up the electronics”), talked about successful completion of a part of their design (“the wood was cut to exactly what we needed”), and displayed continuing high self-efficacy (“I feel pretty confident working by myself;” student A, mid-semester). In contrast, the older student focused on what she could not accomplish (“When we first started, I thought we would make the real thing at the end, but in the end, we [will just] make the model”), and repeatedly discussed how things had turned out differently than she had expected (“I thought you didn't have to do this much research…But, we have to research on this one thing and it took us a really long time;” student B, mid-semester). The older student’s frustration with the project’s progress was also clearly tied to her concerns about time constraints, as previously discussed. Trends in qualitative data also showed that although lower-level class students discussed their concerns with the limited time available, they tended to talk more positively about how they altered their designs to fit the timeframe. In contrast, higher-level class students typically voiced more frustration over what they could not accomplish in the time allotted. We also noted that higher-level class students typically began with more complex, time consuming projects. Many of them ended up building only models of their designs instead of actual design products like they had hoped. The resulting letdown may have influenced the negative outlooks they had of their progress and, relatedly, the decrease in self-efficacy by mid-semester.

### Situational interest trajectory

#### Situational interest declined by the end of semester

To answer the second research question, hierarchical linear growth models were fitted to examine the change in students’ situational interest from the middle to the end of the semester. Standardized scores were used for situational interest, self-efficacy, and achievement emotions, and as such self-efficacy and achievement emotion regression parameters could be interpreted as beta weights. Results from the unconditional model showed that students’ situational interest declined from the middle to the end of the semester (Table 6). The second, control variables-only model showed that class level was a large and significant predictor of situational interest, with the higher-level class associated with lower situational interest at each time point. Gender was not a significant predictor of situational interest. In the full model, self-efficacy scores at the beginning of the semester (self-efficacy_0_) and as a time varying predictor at the middle and the end of the semester (self-efficacy_1,2_) were added to the model. Whereas students’ initial self-efficacy did not significantly predict interest scores, contemporaneous self-efficacy scores were found to significantly predict students’ interest at each time point. For example, students who had higher self-efficacy scores at the mid-semester data collection also tended to report higher makerspace interest at the same time point. The full model also added achievement emotions as predictors, and found that excitement and frustration were significantly associated with situational interest. Interestingly, when we compare these results to the self-efficacy growth model, excitement emerged as a significant predictor for both self-efficacy and situational interest. However, while experiences of confusion are significantly associated with self-efficacy scores, it was found not to be a significant predictor in this model. Instead, frustration was found to be significantly and negatively associated with situational interest. Finally, we fitted interactions between the time variable and emotion variables, as well as between the time variable and self-efficacy, but none of these specified moderation effects were shown to be statistically significant. These interaction effects were then removed, and the previous model (full model) was retained as the final linear growth model of situational interest.

**Table 6:** Hierarchical linear growth model of makerspace interest

	Unconditional model	Controls-only model	Full model
Time_1–2_	− 0.37*** (0.08)	− 0.35*** (0.08)	− 0.32*** (0.08)
Self-efficacy_1,2_	–	–	0.29*** (0.06)
Self-efficacy_0_	–	–	− 0.07 (0.05)
Excited	–	–	0.46*** (0.06)
Curious	–	–	0.05 (0.05)
Frustrated	–	–	− 0.19*** (0.06)
Confused	–	–	0.08 (0.06)
Class level (lower-level class = 0)	–	− 0.93*** (0.16)	− 0.36*** (0.10)
Gender (male = 0)	–	0.05 (0.16)	− 0.03 (0.10)
Intercept	0.67*** (0.16)	0.92*** (0.27)	0.36* (0.15)

Unstandardized regression coefficient (standard error)

Notes: subscripts refers to data collection rounds; 0 = beginning of semester, 1 = mid-semester, 2 = end of semester. Standardized scores were used for interest, self-efficacy, and achievement emotion scores

* *p* < 0.05; ***p < 0.001

**Fig. 3:**
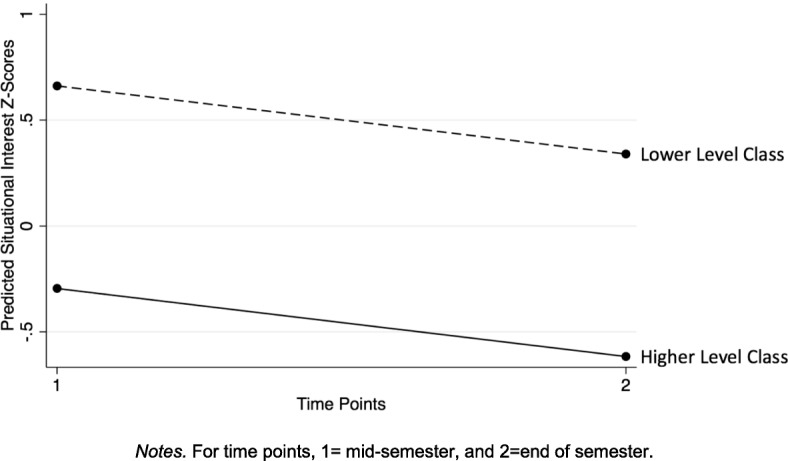
Predicted situational interest trajectories, by class level

Similar to our analysis of self-efficacy growth, we also modeled changes in situational interest for the average male student (gender = 0) in each makerspace class level and graphed these predicted trajectories to examine trends within our specific makerspace context (Fig. 3). Results showed that the average student in the lower-level class (with all motivation scores held at class level means) had more adaptive interest trajectories than students in the higher-level class. This result echoed self-efficacy trends in which students in the lower-level class had higher self-efficacy scores at each time point, as well as a less steep decline in self-efficacy overall.

#### Positive emotions key to interest development

Qualitative data showed that students expressed relatively high levels of interest at the beginning of the semester. Almost all students were able to articulate personal reasons for choosing the problem they wanted to solve with their design project. For example, a boy in the higher-level class who decided to make a prosthetic arm that can be adjusted as the user grows older told us, “So, you don't have to get it replaced every five years, which is very expensive for people. And the average prosthetic cost about 25,000 dollars. So, it's very expensive. I've always been really interested in this. So, I tried to look up problems of people with prosthetics. So, I chose this” (Table 7; student D, beginning of semester). We noted that this student had a classmate who was missing an arm, which may have triggered his interest in prosthetics. Students’ personal ownership of their design project is optimal for triggering situational interest, as corroborated by quantitative descriptive results showing moderately high levels of interest present at the beginning of the semester.

**Table 7:** Exemplary student quotes demonstrating situational interest trends

Class level	Beginning of semester	End of semester
Student team C, (males/lower-level class)	Student 1: I wanted to solve the problem of the drought… we are starting to learn about filtering gray water. Student 2: I wanted to do something else, but I decided I wanted to help solve the drought problem too.	Student 1: In our final project the gray water goes out with a straw with pressure to water the plants, so you do not waste it and you do not need another water source to water the plants. The best part was figuring it all out. Even though we wanted to do something different, we are proud of it. We might use it, we do not know. We have not done the final yet. Student 2: It’s almost a success, we just need to finish it.
Student D, (male/higher-level class)	What we're trying to do is to make these prosthetics move as you grow. Say you were born without an arm. We're trying to make it so that it will grow with you. We will split it in half so you can adjust the size. So, you don't have to get it replaced every five years, which is very expensive for people. And the average prosthetic cost about 25,000 dollars. So, it's very expensive. I've always been really interested in this. So, I tried to look up problems of people with prosthetics. So, I chose this.	This is supposed to be the final product of the middle arm, but apparently I have to make it over again…I don't know. They say that I can't use it. I took a cylinder shape that's already downloaded into the maker box. They said I need to do it again because I need to make it. It needs to be wider, too. I don't know if it needs to be wider because we will be adding a rotor that makes it bend. I don't know if it needs to be wider, I need to talk to people on that… I'm just trying to make everything perfect right now, so I don't have to make it again.

As the semester wore on, students’ interest levels seemed to change to varying degrees. Some students, especially those in the lower-level class, continued to express interest in their design project. For example, a boy in the lower-level class expressed intrinsic enjoyment in working on his project (“The best part was figuring it all out”), pride in their accomplishment despite having to alter their plans to adapt to logistic constraints (“Even though we wanted to do something different, we are proud of it”), positive evaluation of their progress (“it’s almost a success”), and expressed interest in finishing the project in the future (“we just need to finish it;” Table 7, student team C, end of semester). This pattern demonstrated how positive emotions such as enjoyment while working, excitement towards overcoming difficulties, and pride at what was accomplished contributed to maintained situational interest. On the other hand, other students who initially started off with a high level of interest had notably lower interest after encountering repeated unsuccessful trials as part of the design process. This was especially true for many students in the higher-level class who had more complex projects. For example, the same boy in the higher-level class who had expressed high-triggered situational interest towards making a prosthetic arm told us by the end of the semester: “This is supposed to be the final product of the middle arm, but apparently I have to make it over again… I'm just trying to make everything perfect right now, so I don't have to make it again” (student D, end of semester). His attitude was similar to many of his peers, who by the end of the semester expressed their desire to just complete what was required and showed less interest in the success of the outcome. These trends showed that initial triggered situational interest does not necessary translate into maintained interest, especially when students experienced frustrations that arise from the iterative nature of the design process.

Finally, a small group of students displayed very high interest by the end of the semester. For example, a pair of male students in the higher-level class wanted to design an inexpensive watch that could double as a personal heart rate monitor. Like everyone else, they experienced some setbacks. At the mid-semester interview, they recalled when “the LCD was glitching out and [they] just couldn’t figure it out” (quotes not shown in table). However, at that mid-semester think-aloud interview, they experienced an aha moment when their heart rate monitor finally worked. The excitement was palpable. They told to us, “we finally got the light to pulse! It works!” At the end of semester interview, one student in the team told us that although they could not successfully fit the heart rate monitor in a watch prototype in the time they had, they will try to finish it outside of class. He said, “We're going to work over the summer to try to finish it. My dad has a studio, we could just work there.” Here, we saw how a positive emotion like excitement may have enhanced maintained situational interest, so much so that the students had made a choice to continue working on the project even when the makerspace class was over. We noted that this pair of students was in the higher-level class, and their interest level did not match the average pattern for other students in their class. Our qualitative data suggests that interest development is less tied to age per se, but more specifically to the complexity of students’ design project, how much they were able to experience success in fulfilling their design plans given that complexity, and the level of positive and negative emotions they experienced throughout the process.

## Discussion

Our objective was to explore elementary school students’ self-efficacy trajectory through a semester-long makerspace course, and how changes in self-efficacy are associated with experiences of positive and negative achievement emotions. Additionally, we examined students’ situational interest trajectory, and how it is related to self-efficacy and achievement emotions. One important finding of this study is the result that students’ situational interest and self-efficacy remained moderately high overall, with most students scoring above the median score in situational interest and self-efficacy across all time points. In general, students also had more positive emotional reactions to makerspace activities compared to negative emotions. These results echoed the positive motivational benefits of design-based learning found in previous studies. Specifically, the overall moderate to high levels of self-efficacy and situational interest could be due to the increased student autonomy and ownership towards learning, as demonstrated by several studies (Doppelt 2009; Gerber et al. 2012). The present study also suggests that students’ overall positive emotional reactions towards makerspace activities are likely associated with relatively high self-efficacy and interest in the design-based makerspace program. Our findings echoed trends from previous research, which found positive relationships between positive emotions and self-efficacy (Bandura et al. 1996; Carrier 2009; Goetz et al. 2008; Ren 2000) and between positive emotions and interest (Ainley et al. 2002; Hidi and Renninger 2006).

### Self-efficacy and interest decline

However, a closer examination of results from piecewise growth modeling demonstrated that students’ self-efficacy decreased as the makerspace course progressed, and students encountered challenges with completing their projects. The decrease in self-efficacy occurred markedly between the beginning and the middle of the semester, and occurred to a greater extent for students in the higher-level class. We also noted a qualitative trend in our data that older students tended to choose more complex projects that often required more time to complete. As such, many students in the higher-level class were unable to produce an actual design product, but rather a working prototype. Project complexity may have factored into older students’ poor evaluation of their progress and decreased perception of self-efficacy.

This pattern of self-efficacy decrease supports previous findings that students often overestimate their abilities, especially when evaluating their competencies for distal, long-term goals (Bandura and Schunk 1981). Additionally, this self-efficacy trend is characteristic of the problem-solving process students undergo in the design-based makerspace course. According to Jonassen’s (1997, 2000) model of ill-structured problem-solving, students in this makerspace course needed to define the “problem space” by coming to a personal understanding of what solving their specific problem requires. It is likely that the decrease in self-efficacy from the beginning to the mid-point of the semester reflects challenges students encounter with overambitious goals for their projects. Students likely reevaluated their self-efficacy perceptions as they got more concrete ideas of what they needed to do to complete their designs, formed subgoals that are needed, and became more aware of logistic constraints such as the limits of their ability and available time (Bandura and Schunk 1981). Overall, these results point to the need to support students’ self-efficacy in design-based makerspaces, particularly in the beginning phases as students start their projects. Findings from this study also suggest that self-efficacy support may be more effective when coupled with teacher scaffolding in the initial decision-making process, helping students choose projects at the right level of difficulty.

Our study also found that students’ situational interest significantly decreased from the middle to the end of the course. Quantitative data showed that self-efficacy beliefs were associated with students’ situational interest. Qualitative data helped explained this trend, showing that students who had positive evaluations of their progress tended to express more interest in the success of their design projects, and were more likely to display maintained situational interest. This finding empirically supports the relationship between self-efficacy and interest, as conceptualized by social cognitive theory (Bandura 2001; Bandura and Schunk 1981). The close relationship between self-efficacy and interest we found adds to a growing number of empirical studies that have documented this trend (Bandura and Schunk 1981; Linnenbrink and Pintrich 2003; Lee et al. 2014; Cordova et al. 2014). Our results show that even for makerspace activities, which has been touted for their inherently interesting nature, self-efficacy still plays an important role in triggering and maintaining students’ interest.

The present study also contributed additional insights on how self-efficacy may be associated with interest longitudinally. We found that contemporaneous self-efficacy beliefs measured at the same time as situational interest had significant predictive power on interest. In contrast, self-efficacy beliefs assessed at the beginning of the semester was not found to be associated with interest measured some time later. This result from hierarchical linear modeling added a temporal dimension to the relationship between self-efficacy and interest. Specifically, students’ appraisal of ability after task engagement had a more direct relationship to their current interest levels compared to initial appraisal of ability.

The relationship between self-efficacy and interest found in our study extended the theoretical conceptualization of social cognitive theory. Bandura and Schunk (1981) theorized from results of their intervention study that increases in self-efficacy are associated with interest development, but this process occurs over time rather than instantaneously. They suggested that the build-up of mastery experiences over a period of time enhances students’ interest, and there may be a “temporal lag” between self-efficacy increase and interest change (p. 597). In their study, students needed to experience a number of progressive successes as part of the intervention plan before improved interest occured. Similarly, some intervention studies that have induced self-efficacy increase have not always found an immediate relationship between increases in self-efficacy and interest development (Bailey et al. 2017). In contrast, other studies have found that self-efficacy and interest are closely and contemporaneously related (Cordova et al. 2014; Lee et al. 2014; Wigfield et al. 1997). It should be noted that context differed among these previous studies, with some occurring in intervention settings and others in naturalistic settings. Our context differed from an intervention setting in that many students in the makerspace course did not experience progressive successes, but rather repeated setbacks that come with the design process. In our case, we found that a decrease in students’ self-efficacy had a more immediate association with their interest. Also, students’ high self-efficacy at the beginning of the course may not help cushion this interest decline. It may be the case that in naturalistic settings like ours where self-efficacy tended to decline after reappraisal, self-efficacy levels would have a more immediate relationship with interest. This further emphasized the need to support students’ self-efficacy throughout the design process. Our results also pointed to the need for additional research on the temporal relationship between self-efficacy and interest development in different contexts.

### Achievement emotions associated with efficacy and interest trends

Another important finding from this study is the association between achievement emotions and students’ self-efficacy trajectory. Quantitative results showed that experiences of excitement and confusion were related to levels of self-efficacy, with more excitement associated with higher self-efficacy scores and more confusion associated with lower self-efficacy scores. The directional relationship between achievement emotions and self-efficacy echoed previous findings by other researchers (Goetz et al. 2008; Goetz et al. 2006; Pekrun et al. 2006; Gumora and Arsenio 2002). Additionally, we found that students’ experience of excitement in the makerspace program was not only associated with an increase in self-efficacy at each time point (i.e., the intercept), but was also associated with a lower rate of self-efficacy decline (i.e., the slope) from the beginning to the middle of the makerspace course. This points to the particular role positive emotions like excitement plays in students’ self-evaluation of competency. In light of social cognitive theory, our findings make theoretical sense because the experience of positive emotions such as excitement is appraised as an outcome associated with the makerspace project (Bandura 2001; Linnenbrink and Pintrich 2003). During the self-reflection process, students who experienced excitement likely view this emotional outcome as evidence of their progress towards success, lessening the decline of self-efficacy despite other deterrents they may have experienced. Our findings suggest that we need to consider both positive and negative emotions when examining their associations with self-efficacy trajectories.

Achievement emotions were also found to be associated with students’ situational interest. Specifically, quantitative results showed that excitement had a positive association with students’ interest in the makerspace course. These trends were also corroborated by qualitative accounts, where students’ positive emotions were clearly associated with maintaining student interest. Our results are similar to those of previous studies, which have found that positive emotions are closely linked to interest development (Ainley et al. 2002; Hidi and Renninger 2006). In their description of the four phases of interest development, Hidi and Renninger (2006) pointed out that positive emotions like excitement is particularly important for maintaining interest. In our qualitative accounts, students who experienced intense excitement of successfully solving a design issue also exhibited indicators of maintained interest, such as choosing to continue work on the project when it was no longer required. Examining implications of positive emotions on interest development after interest has already been triggered is an important avenue for additional research and would provide more insights into the close relationship between achievement emotions and interest development.

Furthermore, negative emotions were also found to be linked to decreased self-efficacy and interest. Whereas confusion was significantly associated with decreased self-efficacy, frustration was associated with decreased situational interest. One explanation for this associative trend is the fact that confusion may be more related to ability perceptions, and would have a more direct influence on students’ self-efficacy. On the other hand, frustration is a more general negative emotion that may arise as a result of various task-level characteristics, and is thus more related to global interest levels rather than ability beliefs. These results lend additional support to previous studies that have found links between negative emotions and decreased self-efficacy (Gist et al. 1989; Caprara et al. 2008; Ren 2000), and negative emotions and decreased interest (Ainley et al. 2002). However, these studies tended to group negative emotions together to discern their collective association with other motivational factors like self-efficacy and interest. Results from the present study demonstrate that more work is needed to examine specific relationships between different types of negative emotions and other motivational constructs.

Lastly, previous studies have shown that negative achievement emotions like confusion may sometimes promote deep inquiry and effortful thinking, which lead to increased learning (D’Mello et al. 2012; D’Mello et al. 2014). The positive benefits of confusion only occurred when learners had sufficient ability to regulate this negative emotion and were provided with scaffolds to help resolve the confusion. In these past studies by D’Mello et al. (2012, 2014), confusion was experimentally induced, specific scaffolds were given to help resolve confusion, and the samples were undergraduate students. However, in our setting, elementary school students may lack the necessary emotional regulation skills to turn experiences of confusion into learning gains. Further, confusion and frustration were to a certain extent part of the design process, and specific scaffolds were not always given to help students overcome their negative emotions. Also, unlike in experimental contexts, design-based courses are designed to be student-led. Students are asked to lead the learning process, independently seek information needed for design, and figure out how to resolve conflicting information when they experience confusion. In this naturalistic setting, the benefits wrought by negative emotions like confusion need to be carefully balanced with other negative implications such as decreased self-efficacy and interest.

### Promoting self-efficacy and interest in context-specific design-based makerspaces

As suggested by social cognitive theory guiding our study, environmental factors also interact with the motivational processes described above. Context-specific factors play a role in students’ emotional reactions to design-based makerspace tasks, as well as how their self-efficacy and interest change throughout the course. In our study, students were from primarily affluent families and were situated in a well-performing, high-resourced school. In fact, we noted that students in our study had little constraints in terms of accessing the resources they need to complete their design projects. While this access to resources may have supported students’ creativity, it also widened the possibilities in the types of projects students can take on. This contributed to many students undertaking complex projects and spurred disappointment when initial project goals were not reached. Further, students in this specific makerspace program were in a high-performing school and most are used to succeeding in academic tasks. Coming up against the repeated failures as part of the iterative design process may have induced more frustration than usual in this population. Further, students in our study were elementary school students whose understanding of STEM concepts may be rudimentary and who may not be used to engaging in long-term design projects. All of these contextual factors likely played a role in these students experiencing negative emotions and a decline in self-efficacy as they faced difficulties executing their design projects.

The overall takeaway of the present study is the need for instructional interventions in order to ensure that design-based makerspaces constitute a positive and motivating experience for students. Many scholars have touted makerspace as a motivating learning environment. While this is in part true, our study showed that the iterative design process, coupled with context-specific issues such as students choosing overly-complex projects, can lead to suboptimal outcomes on students’ self-efficacy and interest. Makerspace instructors need to provide emotional regulation and self-efficacy support to ensure adaptive self-efficacy and interest development, and this support may vary by context. According to past research in this area, instructors can promote students’ self-efficacy and positive emotions by providing verbal persuasion to convince students of their ability, and small successive goals to maintain self-efficacy levels (Bailey et at. 2017; Bandura 2001; Bandura and Schunk 1981). Our analysis also suggests that students in high-resourced makerspaces like the one highlighted here especially need support in translating their design goals into feasible project objectives. Having too many choices can result in students wanting to take on too much, and instructors need to help students narrow the field of choices into ones that can be successfully completed given students’ ability level and time allotment. Lastly, when working with typically high-performing students, makerspace instructors should explicitly discuss with students that setbacks are a necessary part of the design process and point to real-life models in the STEM field who cultivated failures into success. The goal is to help students understand the reality of the design process, and that failure in this context is expected and not solely reflective of their ability or effort.

It is important to note that students in makerspace programs with different contextual characteristics, such as one within lower-performing schools or with less resources, may need different types of support. While our study suggests that supporting students’ self-efficacy and emotional regulation need to occur across makerspace settings, the types of instructional support that are effective in each context may differ. For example, students in makerspaces with less resources may not need as much help narrowing down the resources they can use or the size of project they can undertake. Perhaps self-efficacy support in that environment may entail completely different strategies, such as providing students with project ideas that fit with available resources. More research is thus needed to understand motivational processes that occur in varied makerspace contexts, and what the right type of support might look like in each case. Makerspaces have the potential to help students learn STEM concepts and trigger students’ interest in STEM. But, the positive benefits of makerspace courses can only occur when students’ self-efficacy and positive achievement emotions are also fostered with instructional strategies that are context-sensitive.

Part of the importance of including STEM tasks such as makerspaces into an academic curriculum is to help students form positive STEM interest. Students who have positive experiences with STEM-related activities develop a more enduring interest in STEM, possibly contributing to their decision to pursue STEM careers later on in their educational trajectory (Lent et al. 2002). Previous research has also shown that perceived competence and interest in performing STEM tasks are closely tied to the development of STEM identity, or students’ perception that who they are is consistent with becoming a member of the STEM community (e.g. becoming a scientist or engineer; Kim et al. 2018). Therefore, the decline in self-efficacy and interest as students participate in design-based makerspaces may be detrimental to the development of STEM identity. It signals to students that they lack the skills and inclinations necessary to become part of the STEM community. Fostering student self-efficacy and interest in STEM-focused makerspaces go beyond motivating students towards the learning task at hand, but also have to do with preparing students to see themselves as someone who can “do STEM” in the future. Educators who are integrating makerspaces into schools need to have this broad view to ensure that the learning and design goals they set do not ultimately interfere with students’ positive development of STEM interest and identity.

## Limitations and future directions

Like any research study, the present study was constrained by contextual and logistic factors. One limitation of this study is the lack of a comparison group to discern whether the declining self-efficacy and interest trajectories we found were typical of students participating in makerspaces. Specifically, we cannot infer that the noted decline in these motivational attributes are due to students’ participation in the design-based makerspace program, as opposed to a reaction to participating in novel learning tasks in general. For instance, it is likely the case that students participating in “traditional” STEM instruction begin the course with even lower self-efficacy and interest, and/or have a higher rate of decline in these motivational attributes. This has been demonstrated in studies examining students’ current attitudes towards learning science in schools (Osborne et al. 2003; Tytler and Osborne 2012). However, a comparison group would have been difficult to define, as the scope of the design-based makerspace course covers multiple subject areas and technical knowledge. Also, the intention of this research is to understand students’ motivational characteristics in a naturalistic makerspace setting. While having a comparison group may have been helpful, it was not a central part of our research aims. An additional limitation is the use of self-report measures for the focal variables of this research, which may lead to self-report bias. The main disadvantage of self-report stems from the fact that people do not always report the truth. There are biases stemming from both limitations in people’s conscious self-knowledge as well as the tendency to align reported behaviors and perspectives with what they view to be socially desirable (Barker et al. 2002; Pintrich 2004). However, we note that the focus of our research is on motivational variables, which could only be gathered via self-report. We also worked to triangulate our quantitative findings by collecting contemporaneous qualitative data through interviews and observations. Understanding complex motivational reactions to STEM-related activities like design-based makerspaces requires integrating results from multiple studies with different methodological lens, suggesting a need for more varied future research in this area.

## Conclusions

Despite these limitations, our study was one of a few that have examined motivational implications of a STEM-focused, design-based makerspace program. The study was also unique because we sought to understand the ways in which makerspace programs influence elementary school students’ motivation, compared to previous studies which have focused on older students’ motivational responses. Overall, results from this study point to the potential for design-based makerspaces to support elementary students’ learning in STEM, albeit with a cautionary note that such experiences should include efficacy- and emotion-related scaffolding. With this motivational support system in place, design-based makerspace programs have the potential to be an effective instructional medium for developing both STEM-related knowledge and interest.
